# Neuroma of the Infrapatellar branch of the saphenous nerve following Total knee Arthroplasty: a case report

**DOI:** 10.1186/s12891-019-2934-0

**Published:** 2019-11-13

**Authors:** Yongbo Xiang, Zeng Li, Peng Yu, Zhibo Zheng, Bin Feng, Xisheng Weng

**Affiliations:** Department of Orthopedic Surgery, Peking Union Medical College Hospital, Peking Union Medical Colleage, Chinese Academy of Medical Science, No.1 Shuaifuyuan, Dongcheng District, Beijing, 10073 China

**Keywords:** Neuroma, Total knee arthroplasty, Complication

## Abstract

**Background:**

Injury to the infrapatellar branch of the saphenous nerve (IBSN) is common during total knee arthroplasty (TKA) with a standard midline skin incision. Occasionally, painful neuromas form at the transection of nerve and cause pain and limitation of the range of motion of the knee joint.

**Case presentation:**

A 70-year-old woman experienced right knee pain and stiffness for 4 years after TKA. Physical assessment revealed medial tenderness; Tinel’s sign was positive. Radiographs revealed that the prosthesis was well-placed and well-fixed. She was diagnosed with arthrofibrosis and possible neuroma after TKA. She underwent right knee exploration, neurectomy, adhesiolysis and spacer exchange. The neuroma-like tissue was sent for pathological examination. The patient recovered uneventfully and at 3-month follow-up reported no recurrence of pain or stiffness. The pathological report confirmed the diagnosis of neuroma.

**Conclusions:**

IBSN injury should be a concern if surgeons encounter a patient who has pain and stiffness after TKA. Tinel’s sign, local anesthetic injection, MRI and ultrasound could help the diagnosis and identify the precise location of neuroma. Surgical intervention should be performed if necessary.

## Background

In the process of total knee arthroplasty (TKA), injury of the infrapatellar branch of the saphenous nerve (IBSN) or its terminal branches is common. This can lead to paresthesia or anesthesia of the medial and anterior part of the knee [1–3]. Occasionally, a painful neuroma forms at the transection of the nerve, causing pain and limitation of the range of motion of the knee joint [2, 3].

The saphenous nerve is the longest cutaneous branch of the femoral nerve. It perforates through the adductor canal between the tendons of the gracilis and sartorius muscles. It divides into the IBSN and supplies the proximal tibia inferior medial to the patella [2, 3]. Numerous researchers have described the anatomy of the IBSN [4–8], which is divided into three branches. The superior branch runs inferior to the distal pole of the patella transversely. The middle branch arises from the superior branch and passes diagonally across the patellar tendon. The inferior branch extends along the medial border of the patellar tendon and terminates at the tibial tubercle [9–11].

Due to its anatomical characteristics, the IBSN is often damaged by compression, infection, trauma, or iatrogenic factors, such as needles or surgery [4, 12–14]. Unintentional IBSN injury caused by surgery has been reported in anterior cruciate ligament reconstruction [5, 7, 8, 15, 16], standard anteromedial knee arthroplasty [5, 7, 14], vascular surgery of the lower extremity [4], and TKA [2–4, 12]. The prevalence of IBSN damage as a postoperative complication in some studies ranges from 0.5 to 53% [10, 17].

Damage to the IBSN leads to sensory comorbidities in most cases. Problems can include loss of sensation, paresthesia, neuralgia or hypersensitivity in the medial infrapatellar area of the lower extremity [10]. Patients might also develop a post-operative neuroma from the transection area of the IBSN or its terminal branches in rare circumstances [7]. Ilfeld et al. reported that up to 9.7% of primary TKA patients and 21% of revision patients experienced pain after surgery, which could be attributed to neuroma formation based on their clinical data [18]. However, the authoritative incidence of knee neuroma after TKA has not been reported.

Although IBSN injury is common, knee stiffness resulting from neuroma in patients after TKA surgery, requiring revision, is rare. In this article, we present a case of a 70-year-old woman who complained of knee pain and stiffness after TKA. Neuroma was diagnosed after surgical exploration and pathological examination. Her pain and rigidity were completely resolved after neurectomy, adhesiolysis and spacer exchange.

## Case presentation

The patient was a 70-year-old female with continued right knee rigidity and pain after right TKA. Four years earlier, she was diagnosed with severe osteoarthritis of the right knee at another hospital, and TKA was performed uneventfully. She recovered well in a couple of months after surgery, but then she began to feel sharp pain in the medial part of the right knee, and as a result experienced a decrease in the range of motion (ROM). In subsequent years, her right knee pain and rigidity gradually increased. Treatments including non-steroidal anti-inflammatory drugs, cortisone injections and physical therapy provided little or no relief. At our clinic, she reported severe pain on the medial genicular region with any movement of the lower limbs and even at rest. The visual analogue scale (VAS) score for her knee pain was 6/10. Her right knee motion was limited to several degrees. Other medical history involved hypertension, hyperlipidemia, and diabetes mellitus for about 10 years.

**Fig. 1:**
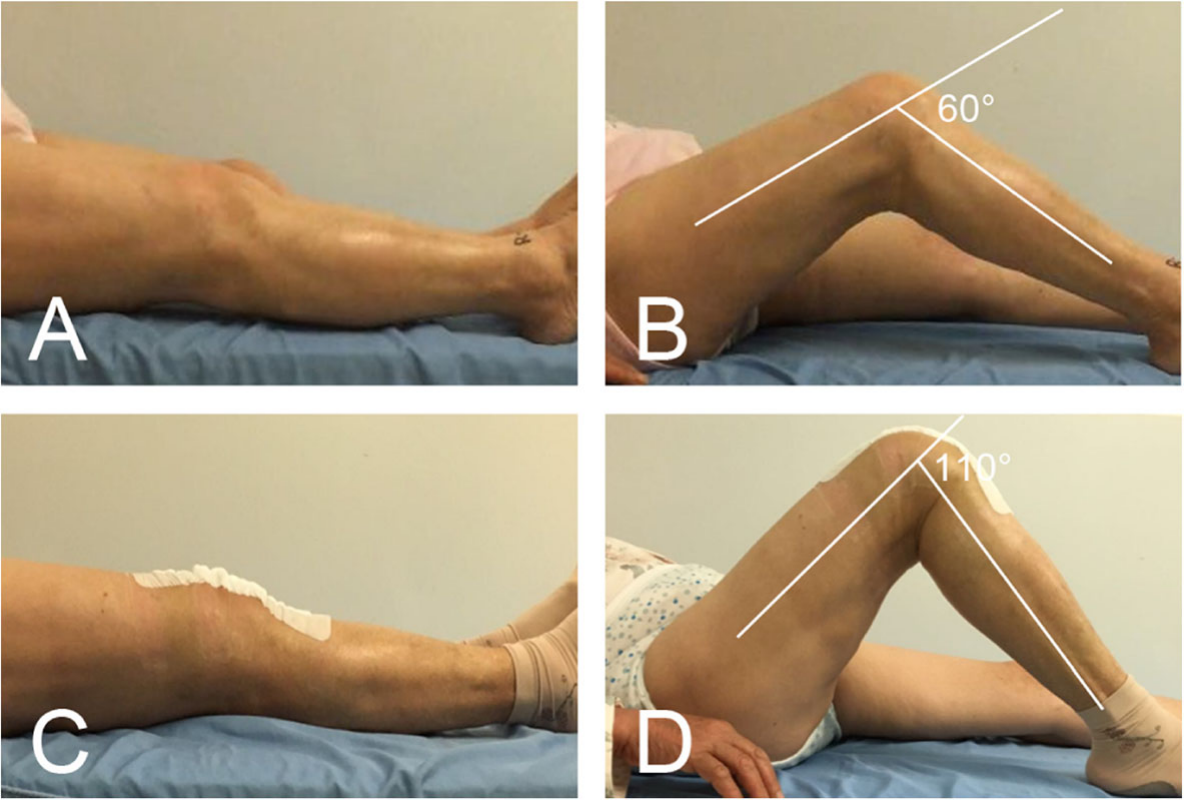
**a**, **b** Patient’s preoperative right knee range of motion (0° to 60°). **c**, **d** Patient’s 2nd postoperative day right knee range of motion (0° to 110°)

On physical assessment, the patient’s BMI was 24.7 kg/m^2^. She walked into the ward with an antalgic and stiff gait. The skin over the right knee surgical incision was well-heeled. There was slight atrophy of her right calf or quadriceps. Tenderness was found at the medial knee compartments but not the lateral compartment. Tinel’s sign was positive, whereas the varus stress test, valgus stress test and patellar compression test were all negative. Active ROM was 0° to 60° (Fig. 1a and b).

**Fig. 2:**
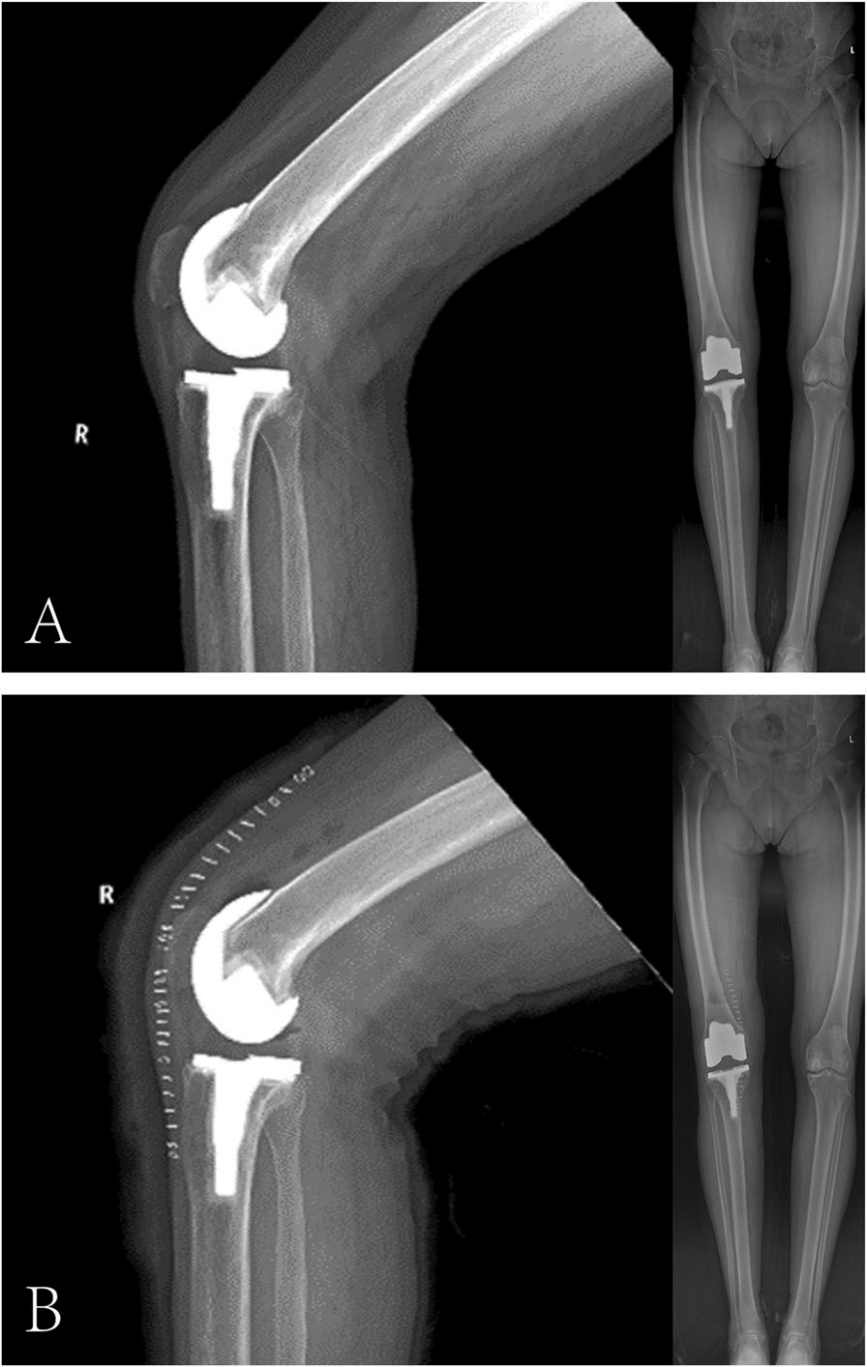
**a** Pre-revision standing anterior lateral radiograph of the right knee joint, revealing that the prosthesis was well-placed and well-fixed. **b** Post-revision radiograph of the right knee joint

Plain radiographs demonstrated that the prosthesis was well-placed and well-fixed without any signs of periprosthetic fracture, implant loosening or osteolysis (Fig. 2a). Magnetic resonance imaging (MRI) was not performed. A diagnosis of arthrofibrosis after TKA was established and neuroma was also highly suspected due to the positive result of Tinel’s sign.

**Fig. 3:**
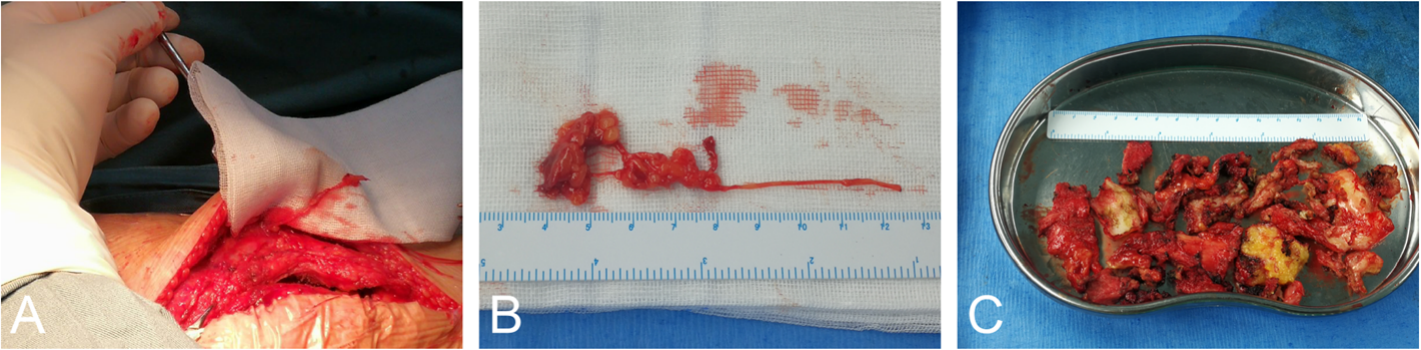
**a** Intraoperative photographs of the medial-sided neuroma. **b** Surgical neuroma specimen that was adhered to nerve, fat and blood vessel. **c** Scar tissue cut by adhesiolysis

The patient then underwent right knee exploration, adhesiolysis and spacer exchange under general anesthesia. Antibiotic prophylaxis was administered for perioperative infection prevention, and the tourniquet was set at 250 mmHg. The surgery was performed through the previous surgical approach, which was a 15-cm standard midline skin incision. After blunt separation of subcutaneous tissue, we observed several thickening neuroma-like tissues distributed in both superior and inferior parts of the medial genicular area (Fig. 3a and b). Based on the innervation around the knee, these abnormal tissues may have been derived from the infrapatellar branch of the saphenous nerve or the medial femoral cutaneous nerve. Some nerve branches were scarred and adhering to surrounding blood vessels and fat in clumps. Subcutaneous neurectomy was performed and the specimen was then sent for pathological examination. The joint capsule was incised through the medial parapatellar approach. Exploration showed hyperplasia of the synovium, and the presence of a large amount of fibrous tissue around the prosthesis, patella and intercondylar fossa. The femoral component and tibial tray showed stable fixation. These findings corresponded to the stiffness of the patient’s physical examination, and confirmed the diagnosis of arthrofibrosis. Adhesion lysis was performed to remove the intracapsular scar tissue (Fig. 3c). Synovectomy was performed to remove hyperproliferative synovial tissue. The polyethylene spacer was replaced by a new one of a suitable size, with a thickness of 11 mm. The incision was then closed, and the patient’s immediate postoperative course was uneventful.

**Fig. 4:**
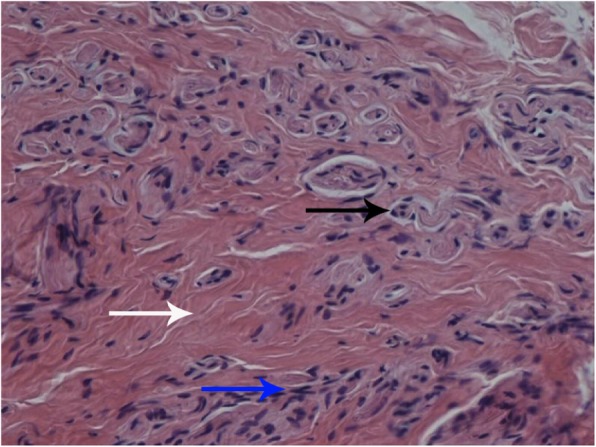
Pathological biopsy of the neuroma showed the haphazard arrangement of nerve fibers. The nerve had been cut both across (black arrow) and longitudinally (blue arrow), and was mixed with fibrous tissue (white arrow)

The patient resumed knee exercise 2 days after surgery and recovered uneventfully. She had complete resolution of right knee pain without pain medication (VAS 2/10 on 2nd postoperative day, VAS 0/10 at 2 weeks postoperatively). The ROM of the right knee was 0° to 110° on the 2nd postoperative day (Fig. 1c and d) and increased to 0° to 120° at 2 weeks postoperatively. The post-revision radiograph revealed similar fixation and placement as pre-revision (Fig. 2b). The pathological report showed that the specimen contained fat, blood vessels, collagen and nerve tissue, and the arrangement of nerve fiber bundles were haphazard, which was considered to be a traumatic neuroma (Fig. 4).

At 3-month follow-up, the patient was mobilizing well without obvious claudication and the ROM of the right knee was 0° to 120°. There had been no recurrence of her right knee pain.

## Discussion and Conclusion

Neuroma of the IBSN after TKA is not a widely known surgical complication. However it has been reported after anterior cruciate ligament reconstruction, arthroscopy, tibial nailing, and TKA [8, 19] and is a cause of chronic pain, even stiffness. There have been several reports in the orthopedic literature [12, 18, 20], but only one of them was pathologically confirmed [2].

We present the case of a 70-year-old female who experienced knee pain and stiffness after TKA. The pain was medial, Tinel’s sign was positive and she reported the VAS score as 6/10. The patient’s resolution of knee pain and recovery of ROM after neurectomy supported our hypothesis about the presence of a neuroma, which was confirmed by further pathological examination. The pain from a neuroma may cause a reduction of joint motion, which then leads to arthrofibrosis and rigidity of the joint. To the best of our knowledge, the present report is the second case to describe a painful IBSN neuroma caused by TKA and confirmed by pathology. Kachar et al. reported the case of a 68-year-old female with knee pain and stiffness caused by neuroma after TKA surgery in 2008. Her symptoms were completely relieved after superficial exploration and resection of the neuroma at 21 months post-operation, but arthrofibrosis was not found and she did not undergo revision [2]. The case reported by Kachar et al. underwent surgery significantly earlier than our case. Our patient did not receive rapid treatment for her postoperative pain, which resulted in long-term movement limitation and arthrofibrosis, therefore revision treatment was inevitable. This suggests that early diagnosis and management is important for patients with postoperative pain after TKA.

Usually, the progress of neuromatous pain is not fast. However, Nagai et al. reported the case of a 74-year-old female who complained of pain just 1 week after TKA, and a positive Tinel’s sign was elicited in the IBSN region. She underwent partial denervation of the IBSN 3 months after TKA, and pain was dramatically relieved. Pathological examination revealed that due to the short duration of the disease onset, the patient had not developed a true neuroma [21].

It is difficult to prevent injury to the IBSN following TKA. Ideally, the surgical incision of TKA should be parallel to the IBSN when technically possible. However a study performed by Kerver et al. found that the location of the IBSN is highly variable, and a safe zone is therefore difficult to define before incision [22]. A recent cadaveric study by Lee et al. claimed that there was no consistent way to preserve the IBSN using a standard midline approach in TKA and patient education on this complication is therefore important [23].

There are several methods that may be helpful in diagnosing neuromas, such as local anesthetic injection and MRI. The pain and stiffness would be relieved if the symptoms were induced by neuroma [2, 3]. Neuroma nodules can also be found on MRI in certain cases. Currently, the precise location of the IBSN neuromas and optimal treatment target area can be identified by Tinel’s test. Some techniques such as use of an ultrasound-guided needle [24] or a peripheral nerve stimulator [25] are also helpful for targeting. With the development of ultrasound technology, high-resolution ultrasound has been used to accurately measure cutaneous nerves. Researchers have been able to find enlargement by measuring the nerve cross-sectional area of certain cutaneous nerves, such as the suprascapular nerve. Therefore, it should also be possible to use high-resolution ultrasound to measure the cross-sectional area of the IBSN before surgery. An early diagnosis and precise location of IBSN neuroma could therefore be achieved [26, 27].

Conservative management of neuromas includes local injection of analgesics, corticosteroids and physical therapy. Surgery is required if conservative treatment fails [12]. Surgical treatment includes IBSN neurolysis, cryoneurolysis [28], neurectomy and selective knee denervation. Worth et al. demonstrated that neurectomy provided better pain relief than neurolysis in patients with saphenous nerve entrapment [29]. Dellton et al. described selective knee denervation as an effective treatment for neuroma pain [30]. Saphenous or IBSN neurectomy or denervation could be performed after diagnosis and targeting. To prevent the recurrence of IBSN neuroma, nerve end cauterizing using an electrocautery might be performed [2].

Regarding the prognosis of patients with neuroma after TKA, early diagnosis and treatment are of great significance. In this case, the patient’s knee pain was not diagnosed and treated in the early stage after TKA, resulting in gradually increasing stiffness and arthrofibrosis. In the end stage, subcutaneous neurectomy alone was not enough to relieve symptoms, and revision was performed to remove the proliferating fibrous tissue and exchange the spacer. We recommend that if surgeons encounter patients with apparent knee pain after TKA surgery, neuroma should be considered as one possible cause as early as possible. Tinel’s test and ROM change after local anesthetic injection could help in distinguishing IBSN injury from intra-articular problems. Surgical intervention should be considered if conservative treatment is not effective.

In conclusion, we recommended that surgeons should be concerned about IBSN injury if they encounter a patient who has pain and stiffness after TKA. Tinel’s test, local anesthetic injection, MRI and high-resolution ultrasound could help to distinguish the etiology of pain and stiffness. Surgical intervention should be performed if necessary.

## Data Availability

The datasets used are available from the corresponding author on reasonable request.

## Abbreviations

IBSN: Infrapatellar branch of the saphenous nerve
ROM: Range of motion
TKA: Total knee arthroplasty
VAS: Visual analogue scale
